# The emerging role of CRISPR-Cas systems in helicobacter pylori and the gut microbiota as regulators of gastrointestinal cancer risk

**DOI:** 10.3389/fimmu.2026.1889115

**Published:** 2026-10-05

**Authors:** Muqadas Bhatti, Husna Irfan Thalib, Ammarah Tayyebah, Aleena Zobairi, Nada Dahan, Fatma E. Hassan

**Affiliations:** 1 Department of Public Health, Bahria University, Karachi, Pakistan; 2 General Medicine Practice Program, Batterjee Medical College, Jeddah, Saudi Arabia; 3 School of Medicine, Cardiff University, Cardiff, United Kingdom; 4 Medical Physiology Department, Kasr Alainy, Faculty of Medicine, Cairo University, Giza, Egypt; 5 Department of Physiology, General Medicine Practice Program, Batterjee Medical College, Jeddah, Saudi Arabia

**Keywords:** biomarkers, tumor, CRISPR-Cas systems, dysbiosis, gastrointestinal neoplasms, *Helicobacter pylori*, microbiota

## Abstract

Gastrointestinal cancers contribute to 26% of global cancer incidence and 35% of deaths, with major contributors such as *Helicobacter pylori (H. pylori)* infection, gut dysbiosis, and lifestyle factors. CRISPR-Cas systems, while best known for their role in bacterial adaptive immunity, also shape bacterial physiology and host–microbe interactions, yet their influence on gastrointestinal carcinogenesis is poorly understood. This review brings together current evidence on the functions of CRISPR-Cas systems in *H. pylori* and the gut microbiota, highlighting their potential impact on cancer risk, diagnostics, and therapy. Key themes synthesized in this review include their role in bacterial immunity, contributions to microbial imbalance and virulence regulation, and emerging applications in cancer detection, microbiome modulation, and precision oncology. CRISPR profiles are being explored as biomarkers, while engineered systems show promise in targeted editing, phage therapy, and highly sensitive diagnostic platforms such as Cas12a-based assays. Despite this progress, challenges remain, including the risk of off-target effects, technical difficulties in detecting CRISPR signatures within complex microbial communities, and a lack of longitudinal human studies. Overall, CRISPR-Cas systems are emerging as natural regulators of microbial ecology and as engineered tools with potential applications in gastrointestinal cancer research, diagnostics, and precision microbiome interventions.

## 1 Introduction

Gastrointestinal (GI) cancers account for 26% of global cancer incidence and 35% of cancer-related deaths worldwide (1). The five major types - colorectal, gastric, liver, esophageal, and pancreatic - exhibit geographic variation in burden (2). Liver and pancreatic cancers have risen in some high-income countries (1), with liver cancer showing the highest increase in incidence and mortality in the United States from 2000 to 2021, followed by pancreatic cancer (3). Globally, colorectal cancer (CRC) has overtaken stomach cancer as the most burdensome GI cancer, though stomach cancer remains the predominant GI cancer in China (4). Key risk factors for GI cancers include advanced age, male gender, family history, smoking, alcohol consumption, obesity, and diets high in red meat (3, 5–7). Chronic infections such as *Helicobacter pylori (H. pylori*), hepatitis viruses, and Epstein-Barr virus also contribute to carcinogenesis (6, 7), while a vegetable-rich diet appears protective (5, 8).

The gut microbiota plays a crucial role in CRC development through various mechanisms, including toxin-mediated deoxyribonucleic acid (DNA) damage and chronic inflammation (9). An imbalance in microbial communities—known as microbial dysbiosis—can result from antibiotics, diet, or infections and is a recognized driver of esophageal, gastric, and colorectal cancers (10, 11). In CRC specifically, dysbiosis may promote tumor development via microbial metabolites, inflammatory responses, and changes in the immune microenvironment (12). *H. pylori*, a major risk factor for gastric cancer, may also influence CRC development by disrupting gut microbial composition and modulating host immunity, potentially altering bacterial colonization in the large intestine (13, 14). Addressing these disruptions, promising interventions such as dietary regulation, prebiotics, probiotics, and fecal microbiota transplantation (FMT) are being explored to restore microbial balance and reduce cancer risk (12).

CRISPR-Cas systems, primarily known for adaptive immunity in prokaryotes, exhibit remarkable evolutionary plasticity and functional versatility (15). Beyond defense against foreign genetic elements, these systems regulate various cellular processes, including virulence, biofilm formation, and quorum sensing (16), with their activity modulated by lineage-specific factors and environmental conditions like stress and growth rate (17). The fundamental mechanism of ribonucleic acid (RNA)-DNA or RNA-RNA complementarity underlies both immune functions and alternate physiological roles (15, 16). This adaptability has led to widespread use of CRISPR-Cas systems in biotechnology and gene editing, with ongoing discoveries of novel types expanding their potential for precision genome medicine (16).

This narrative review addresses the following key questions: (1) What evidence supports a role for endogenous CRISPR-Cas systems in regulating *H. pylori* virulence and genomic plasticity, and what, if any, evidence links these systems to GI carcinogenesis? (2) How do CRISPR-Cas systems influence gut microbial ecology, dysbiosis, and host–microbiota interactions associated with GI cancer risk? (3) What is the current evidence supporting CRISPR-based approaches as biomarkers and therapeutic tools for GI cancers? (4) What are the major knowledge gaps and future research priorities required to distinguish causal from correlative relationships between CRISPR-mediated microbial activity and GI carcinogenesis? By addressing these questions, this review aims to provide a comprehensive overview of both the biological and translational significance of CRISPR-Cas systems in GI cancer research.

### 1.1 Literature search strategy

An organized literature search was carried out to find articles relevant to *H. pylori*, CRISPR-Cas system, gut microbiome, microbial metabolites, and GI carcinogenesis. Literature search was carried out in PubMed, PubMed Central, clinicaltrials.gov, and Google Scholar from inception to 3rd April 2026. The search strategy employed keyword and MeSH term search such as “*Helicobacter pylori*,” “CRISPR-Cas,” “CRISPR,” “anti-CRISPR,” “gut microbiome,” “microbial metabolites,” “short chain fatty acids,” “gastric cancer,” “colorectal cancer,” and “gastrointestinal cancer,” employing Boolean operators (AND/OR). Articles relevant to the purpose of the review were found, giving emphasis to peer-reviewed original research articles, systematic reviews, meta-analysis, and authoritative review articles. Publications not related to *H. pylori*, CRISPR-Cas system, gut microbiome ecology, microbial metabolites, or GI carcinogenesis, or those being duplicates, non-peer-reviewed, and non-English publications were excluded. The references of relevant articles were also scanned to find other relevant articles.

## 2 CRISPR-Cas systems: mechanisms and biological roles

The adaptive immunity mechanisms of bacteria and archaea, known as CRISPR-Cas, comprise a sophisticated mechanism that incorporates foreign nucleic acid fragments, primarily from mobile genetic elements (MGE), into CRISPR arrays that are integrated into microbial genomes. These are classified into two groups on the basis of the architecture of their effector complex. Class 1 (Types I, III, and IV) uses multi-subunit protein complexes for target recognition and DNA cleavage. By contrast, class 2 (Type II, V, VI) use a single, bulky effector protein (e.g. Cas9, Cas12) for efficient interference. This architectural contrast affects their frequency and role in microbial communities such as the gut microbiota, where they mediate genetic exchange and genome integrity, and in turn, host–pathogen interplay and gut health. Additionally, the simplicity of class 2 has made breakthrough genome-editing technologies possible (18).

The working model of CRISPR-Cas immunity consists of three steps. Upon acquisition of a new spacer, which is a fragment of foreign DNA, the Cas1-Cas2 complex integrates it next to the host CRISPR array. The array is then transcribed and matured into short energy-guided CRISPR RNAs (crRNAs) that encode complementary spacer sequences. Lastly, it is in target interference that these crRNAs carry Cas effector proteins (e.g., Cas9, Cas12) to complementary sequences in invading genetic elements, causing precise cleavage and inactivation (19). CRISPR-Cas system has a broad range of functions other than its known role in adaptive immunity. It is a potent repressor of bacterial virulence, frequently acting through the repression of immunostimulatory proteins to evade host immunity. The system heavily influences horizontal gene transfer (HGT), and commonly acts to restrict the dissemination of plasmids and other MGE, which counts for the resistance gene acquisition (20, 21).

The accumulation of these distinct CRISPR-Cas functions is also evident in certain bacteria species. In *H. pylori*, for instance, an orphan CRISPR-like array located within the variant lipoprotein C *(vlpC)* virulence gene exhibited a variable spacers content, which constituted a genetic signature that allowed for strain discrimination at a high resolution, thereby underlying the strong connection between CRISPR elements and pathogen epidemiology (22). A study of *Bacteroides fragilis* further showed highly distinct CRISPR array architectures that could maintain themselves or acquire new spacers with remarkable rapidity (23).

## 3 *H. pylori* and GI carcinogenesis

*H. pylori* is a microaerophilic Gram-negative bacterium whose persistence in the gastric mucosa is enabled by multiple virulence factors that disrupt epithelial integrity and modulate host immunity. The CagA protein, delivered via the type IV secretion system, undergoes phosphorylation and interferes with Src Homology 2 domain-containing Phosphatase 2 (SHP2)/Extracellular Signal-Regulated Kinase (ERK), Phosphoinositide 3-Kinase (PI3K)/Protein Kinase B (AKT), and β-catenin signaling, driving epithelial proliferation and pro-inflammatory gene expression (24–26). VacA induces vacuolation, mitochondrial injury, and T-cell suppression, while urease neutralizes gastric acid and releases ammonia, contributing to mucosal injury (27–29). Adhesins such as BabA and SabA promote colonization, and enzymes like catalase and neutrophil-activating protein (NAP) protect against oxidative stress. Together, these factors activate nuclear factor kappa B (NF-κB) and stimulate interleukin (IL)-8 secretion, sustaining neutrophil recruitment and chronic inflammation (24, 27, 30).

Persistence of *H. pylori* infection leads to the Correa cascade of gastric cancer progression, starting from normal gastric mucosa and progressing through chronic active gastritis, atrophic gastritis, intestinal metaplasia, dysplasia, and eventually gastric adenocarcinoma (**Figure 1**). Bacterial infection and inflammation cause epithelial injury and regenerative processes, while cytokines, pathogenic mechanisms of the bacterium such as CagA and VacA, and oxidative and nitrosative stress contribute to DNA damage, genomic instability, and disturbances in epithelial signaling. Progressive loss of gastric glands and intestinal-type cellular changes create a microenvironment conducive to dysplasia and malignant transformation.

**Figure 1:**
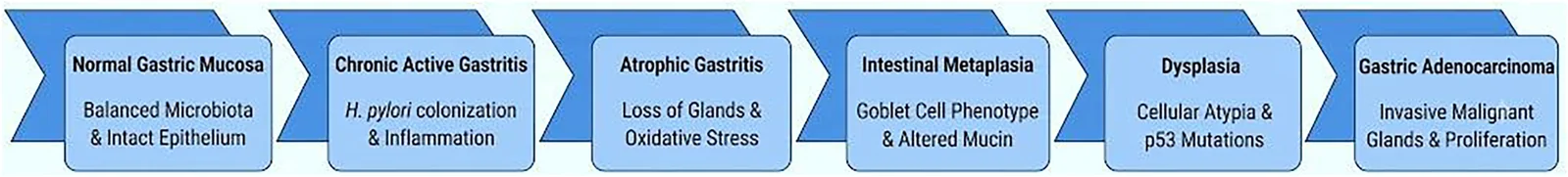
Correa cascade of gastric carcinogenesis. Progressive sequence from normal gastric mucosa to gastric adenocarcinoma.

Persistent cytokine production, including IL-6, IL-8, and tumor necrosis factor-α (TNF-α), maintains this inflammatory environment. CagA disrupts E-cadherin junctions and activates epithelial–mesenchymal transition (EMT) transcription factors (Snail, Slug, and ZEB), enhancing invasiveness (30–32). VacA, along with reactive oxygen and nitrogen species generated by inflammatory cells, induces mitochondrial dysfunction and DNA damage, including 8-oxoG lesions and double-strand breaks. Impaired repair mechanisms further amplify genomic instability (33–35). Oncogenic pathways such as ERK, AKT, Wnt/β-catenin, signal transducer and activator of transcription 3 (STAT3), and Hippo/YAP become dysregulated, while autophagy and endoplasmic reticulum (ER)-stress responses modulate the survival of pre-malignant cells (31, 35).

A hallmark of *H. pylori* biology is its genetic plasticity. The bacterium is naturally competent, acquiring exogenous DNA through the comB-encoded transformation system, with competence genes like *comH* essential for uptake (36, 37). High recombination and mutation rates rapidly diversify virulence factors, outer membrane proteins, and antibiotic resistance determinants. Mosaic forms of the cag pathogenicity island and variable *vacA* alleles are linked to differences in host inflammation and cancer risk across populations (36). **Table 1** summarizes the major *H. pylori* virulence factors, their underlying mechanisms, and associated pathological consequences.

**Table 1:** *Helicobacter pylori* virulence factors, genomic features, mechanisms of action, and pathophysiological consequences (22, 24–37, 61, 82, 83).

Virulence factor/feature	Mechanisms of action	Pathophysiological consequence
A. Established/conventional virulence factors		
CagA	Delivered into gastric epithelial cells via the type IV secretion system; interferes with SHP2/ERK, PI3K/AKT, and β-catenin signaling and disrupts E-cadherin junctions.	Promotes epithelial proliferation, epithelial–mesenchymal transition (EMT), pro-inflammatory signaling, and increased cellular invasiveness, contributing to gastric carcinogenesis.
VacA	Induces cellular vacuolation and mitochondrial injury and suppresses T-cell responses.	Epithelial damage, impaired cellular viability, and immune evasion, facilitating persistent infection.
Urease	Neutralizes gastric acid and releases ammonia, enabling survival within the acidic gastric environment.	Gastric mucosal injury and persistent gastric colonization.
BabA	Adhesin that promotes attachment of *H. pylori* to gastric epithelial cells and facilitates colonization of the gastric mucosa.	Enhanced bacterial colonization and persistence within the gastric mucosa, supporting chronic infection and inflammation.
SabA	Adhesin that promotes bacterial colonization of the gastric mucosa.	Sustained gastric colonization and contribution to chronic host–microbe interactions and inflammation.
Catalase	Enzyme that contributes to protection of *H. pylori* against oxidative stress generated during host inflammatory responses.	Enhanced bacterial survival in the oxidative gastric inflammatory environment and persistence of infection.
NAP	Protects against oxidative stress and contributes to host inflammatory responses, including neutrophil recruitment and activation.	Sustained neutrophilic inflammation and chronic gastric mucosal injury.
ROS/RNS	Reactive oxygen and nitrogen species are generated by inflammatory cells during persistent *H. pylori* infection.	Oxidative and nitrosative DNA damage, including 8-oxoG lesions and double-strand breaks, contributing to genomic instability and carcinogenesis.
comB system	Mediates natural competence and high-frequency uptake of exogenous DNA; *comH* is essential for natural transformation.	Genetic plasticity, virulence diversification, recombination, and acquisition or dissemination of antibiotic-resistance determinants.
B. CRISPR-related genomic features and engineered applications		
Orphan CRISPR arrays (endogenous genomic feature)	CRISPR-like arrays, including those within the *vlpC* region, may provide strain-specific genetic signatures and potentially influence bacterial gene regulation.	High-resolution strain discrimination and potential relevance to bacterial adaptation; functional effects on virulence and carcinogenesis remain uncertain.
CRISPR-Cas diagnostics (engineered application)	Cas12a-based systems enable targeted detection of *H. pylori* nucleic acids.	Rapid, sensitive, and non-invasive detection of *H. pylori*, with potential applications in future diagnostic and therapeutic strategies.

CagA, cytotoxin-associated gene A; VacA, vacuolating cytotoxin A; BabA, blood-group antigen-binding adhesin; SabA, sialic acid-binding adhesin; NAP, neutrophil-activating protein; ROS, reactive oxygen species; RNS, reactive nitrogen species; comB, competence gene B system; comH, competence gene H; CRISPR, clustered regularly interspaced short palindromic repeats; Cas, CRISPR-associated protein; SHP2, Src homology 2 domain-containing phosphatase 2; ERK, extracellular signal-regulated kinase; PI3K, phosphoinositide 3-kinase; AKT, protein kinase B; EMT, epithelial–mesenchymal transition; 8-oxoG, 8-oxoguanine.

## 4 Gut microbiota, dysbiosis, and GI cancers

The human gut microbiota comprises a diverse range of bacteria, viruses, and fungi, which play a key role in maintaining epithelial integrity and mucosal immune homeostasis. A balanced gut microbial ecosystem contributes to nutrient metabolism, protection of the intestinal barrier, and modulation of systemic immunity (8). Key commensals such as Bifidobacterium, Lactobacillus, and *Faecalibacterium prausnitzii* promote anti-inflammatory pathways and epithelial repair. Disruption of this balance termed dysbiosis has been increasingly implicated in GI tumorigenesis (38).

Gut microbial dysbiosis can create a persistent microenvironment that supports GI carcinogenesis by disrupting epithelial homeostasis and promoting tumor-promoting conditions. These alterations may interact with host inflammatory responses and epithelial integrity, contributing to the initiation and progression of malignant transformation (**Figure 2**). Chronic low-grade inflammation is a well-established driver of epithelial transformation, with microbial products such as lipopolysaccharide (LPS) and flagellin activating Toll-like receptors and NF-κB pathways, resulting in pro-inflammatory cytokine production and promotion of a tumor-permissive microenvironment (39). Additionally, certain bacteria species harbor genotoxic potential; for instance, enterotoxigenic Bacteroides fragilis (ETBF) secretes BFT toxin, which induces DNA strand breaks. pks+ strains of *Escherichia coli* produce colibactin, a mutagenic metabolite that alkylates DNA and promotes colorectal carcinogenesis (40, 41).

**Figure 2:**
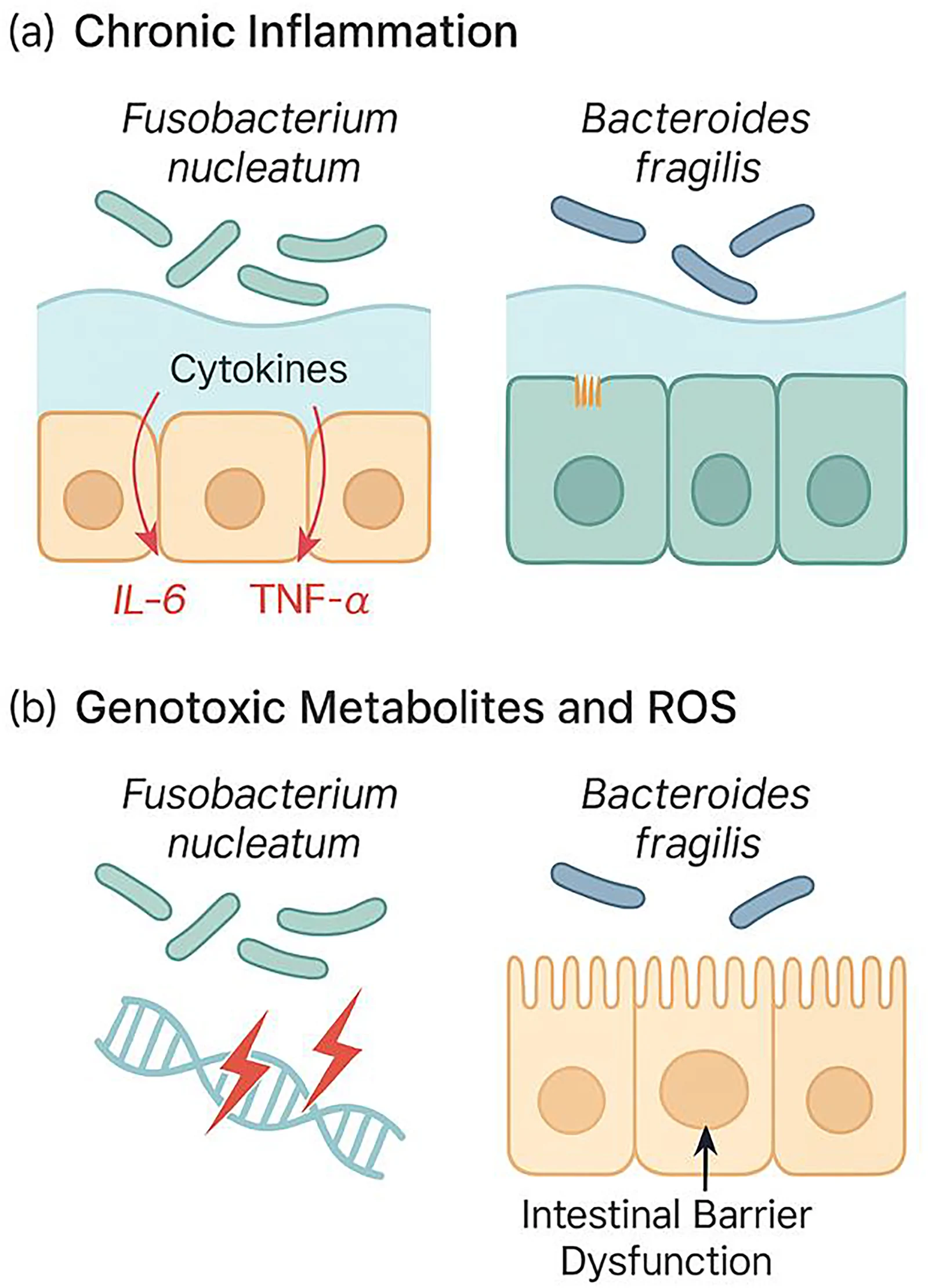
Dysbiosis-driven carcinogenesis. Gut microbial imbalance contributes to gastrointestinal cancer through **(a)** chronic inflammation, where species such as *Fusobacterium nucleatum* and *Bacteroides fragilis* stimulate cytokines (e.g., IL-6, TNF-α), and **(b)** genotoxic effects, where microbial metabolites and reactive oxygen species (ROS) induce DNA damage and intestinal barrier dysfunction.

Furthermore, dysbiosis weakens the mucosal barrier, increasing epithelial permeability and facilitating microbial translocation. This change worsens inflammation and also increases exposure of intestinal stem cells to microbial genotoxins, accelerating oncogenic mutations (42). Specific microbial taxa have also been identified as key players in GI cancers. *Fusobacterium nucleatum* is strongly associated with CRC, enhancing tumor growth via modulation of E-cadherin/β-catenin signaling and inhibition of anti-tumor immunity (43). *Parvimonas micra, Streptococcus gallolyticus*, and *Peptostreptococcus anaerobius* have also been found enriched in CRC tissue. In contrast, *H. pylori*, a well-established gastric carcinogen, promotes tumorigenesis through chronic inflammation, CagA-mediated cellular signaling disruption, and epithelial injury (44). Importantly, *H. pylori* infection alters the composition of the broader gut microbiota, contributing to systemic immune dysregulation and possibly increasing CRC risk in distal sites via inflammatory and metabolic crosstalk (45). Infection reduces microbial diversity in the stomach and small intestine, impairs the production of short-chain fatty acids (SCFAs), and facilitates the overgrowth of pro-inflammatory pathobionts (**Table 2**). Conversely, a dysbiotic gut may impair immune responses to *H. pylori*, promoting persistent infection and carcinogenesis (43–45).

**Table 2:** Pathophysiological mechanisms linking dysbiosis to GI carcinogenesis (39, 40, 43–45).

Dysbiosis feature	Pathogenic outcome	Examples of involved microbes	Molecular/cellular mechanism
Loss of commensals (e.g., *Bifidobacteria*)	Reduced SCFAs production → less butyrate	*Lactobacillus*, *Bifidobacterium* (↓)	Butyrate inhibits HDAC and promotes apoptosis; its absence weakens epithelial defense
Overgrowth of pathobionts	Induction of pro-inflammatory cytokines	*F. nucleatum*, *ETBF*, *E. coli* (pks^+^)	Stimulate IL-6, IL-8, TNF-α via TLR and NF-κB pathways
Genotoxin production	Direct DNA damage	*E. coli* (colibactin), *ETBF* (BFT), *F. nucleatum*	Colibactin alkylates DNA, BFT cleaves E-cadherin → chromosomal instability
Increased intestinal permeability	Immune activation, microbial translocation	*F. nucleatum*, *P. anaerobius*	Leaky tight junctions → LPS entry → systemic inflammation
Chronic inflammation	Tumor-promoting environment	Multiple (especially *F. nucleatum*, *H. pylori*)	Sustained IL-6, IL-8, TNF-α release → cell proliferation and angiogenesis
Immune suppression in tumor microenvironment	Immune evasion by tumor cells	*F. nucleatum* (Fap2 binds TIGIT)	Inhibits NK/T cell activity, promotes M2 macrophages and regulatory T cells

SCFAs, short-chain fatty acids; HDAC, histone deacetylase; ETBF, enterotoxigenic bacteroides fragilis; BFT, B. fragilis toxin; pks^+^, polyketide synthase gene cluster; IL-6, interleukin-6; IL-8, Interleukin-8; TNF-α, tumor necrosis factor-alpha; TLR, toll-like receptor; NF-κB, nuclear factor kappa-light-chain-enhancer of activated B cells; DNA, deoxyribonucleic acid; LPS, lipopolysaccharide; NK cell, natural killer cell; T cell, T lymphocyte; TIGIT, T cell immunoreceptor with Ig and ITIM domains.

## 5 CRISPR-Cas systems in *H. pylori*

Although *H. pylori* is not known to possess a fully functional endogenous CRISPR-Cas system, current research has identified the presence of CRISPR-like loci within its genome, particularly within the *vlpC* gene region (22). These elements should be distinguished from a complete CRISPR-Cas system, as the presence and functionality of associated Cas proteins and CRISPR-mediated interference remain uncertain. The reported *vlpC*-associated CRISPR array has primarily been investigated as a variable genomic feature for strain discrimination (22). Some *Helicobacter* species also harbor type II-C CRISPR-Cas systems or encode virulence-associated protein D (VapD), which shares homology with the Cas2 protein (46). These systems function as adaptive immune defenses by targeting and degrading invading phage DNA and MGEs, thereby limiting HGT and the spread of foreign DNA or antibiotic resistance genes within microbial communities (16, 46, 47) (**Figure 3**). Conversely, anti-CRISPR proteins can inhibit CRISPR-Cas activity and thereby influence the balance between bacterial defense and mobile genetic element dissemination. By interfering with CRISPR-Cas components or their activity, anti-CRISPR proteins may facilitate phage infection and horizontal gene transfer, potentially affecting the acquisition and spread of antibiotic-resistance determinants (47).

**Figure 3:**
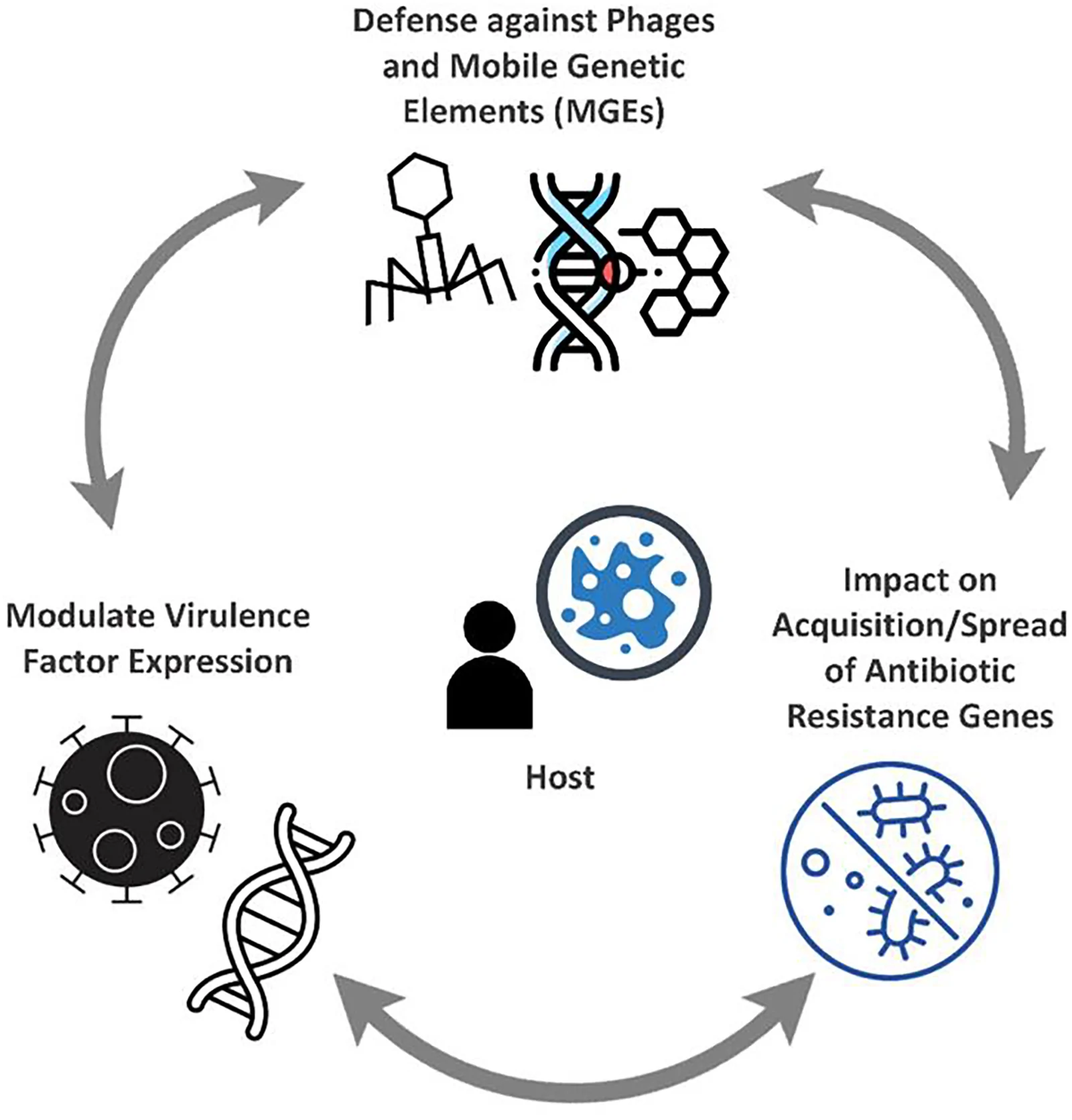
Functional roles of CRISPR-Cas systems. CRISPR-Cas systems contribute to bacterial defense, virulence-associated functions, and antibiotic-resistance dynamics.

Beyond their established role in defense in bacteria possessing functional CRISPR-Cas systems, CRISPR-associated elements have been investigated for potential regulatory effects on endogenous bacterial genes. In *H. pylori*, however, evidence for a functional endogenous CRISPR-Cas system and its direct regulatory effects remains limited. CRISPR-like loci identified in *H. pylori*, particularly within the *vlpC* region, have primarily been investigated as genomic markers for strain discrimination rather than as demonstrated determinants of virulence or cancer risk (16, 46). While the precise regulatory mechanisms are still being elucidated, partial complementarity between crRNAs and host gene sequences appears to play a key role (16).

The CRISPR-like loci have been detected across geographically diverse strains (22). In the Americas, *H. pylori* strains show complex admixture patterns, with distinctive genomic fingerprints for each region. Notably, virulence genes in American *H. pylori* populations exhibit strong fixation and unique evolutionary paths, potentially contributing to the high gastric cancer risk in these regions (48). Comparative genomic studies further reveal significant strain-specific variations in *H. pylori* virulence and antibiotic resistance. In Colombia, a high-prevalence region (80%), analysis of 221 genomes identified two novel clades, diverse virulence genotype combinations, and multidrug resistance profiles in 14.4% of strains (49). In contrast, Sri Lanka shows a very low infection prevalence (1.7%), and genomic analysis of six isolates found an 83.3% rate of metronidazole resistance, but low virulence potential, as evidenced by the absence of key genes like cag pathogenicity island (cagPAI) (50). These findings underscore that geographic strain differences significantly influence infection burden and highlight the need for region-specific, CRISPR-based diagnostic and therapeutic strategies for strain typing and targeted interventions.

## 6 CRISPR-Cas and gut microbiota modulation

CRISPR systems are major contributors to microbial community stability and phage-gene dissemination. They have been proposed to be favored in low-density marine taxa, where they prevent phage-based population crashes, and in human oral sites of high diversity, where they might promote immunity. Nevertheless, the inhibitory effect of CRISPR in HGT of MGE suggests that CRISPR is also a barrier to gene flow, which modulates prokaryotic genome evolution (51). Such dual nature, of allowing as well as constraining gene flow, emphasizes the paradoxical nature of the effect CRISPR has on the evolutionary dynamics and stability in microbial communities.

This transformative effect has immediate relevance for host health, especially in the densely populated gut, hence these systems are an important component of phage/bacteria interaction in the gut microbiome. They act efficiently in regulating phage populations, almost always reducing levels of lytic phages without achieving total elimination (52). A study states that, though CRISPR-Cas does give some degree of protection, it offers only partial immunity in the gut and evidently did not prevent the stable coexistence of *Eggerthella lenta* and its phage in gnotobiotic mice (53). Bacteriophage-carried CRISPR-Cas systems also hold promise for treatment of multiresistant gut microbiome pathogens, re-sensitizing them to antibiotics (54).

The specificity of the CRISPR-Cas and the ability to target the different members of complex microbial communities makes these systems well suited to disrupt carcinogenic microbiome. These systems allow researchers to identify, manipulate, and annotate specific DNA and RNA sequences across diverse cellular contexts paving the way for discovering oncogenes and tumor suppressor genes, mechanisms for drug resistance, and new cancer therapies. This approach has demonstrated potential to eradicate drug resistant bacteria whilst preserving commensal microbiota. However, issues with off-target effects and delivery methods still need to be addressed (55, 56). A study designed an inducible CRISPR-FnCas12a base editor system for effective and programmable genetic editing in human gut *Bacteroides* strains. It makes markerless deletion of bulky genome region (up to 50 kb) possible like of metabolic gene cluster, and insertion of reporter gene. This kit permits the functional analysis of particular bacterial genes and pathways, providing a practical approach to commensal bacteria engineering. This could be applied to reduce the potency of pathobionts, lowering pro-carcinogenic pathways or strengthen the protective capabilities of commensals, with the goal of selectively altering the microbiome to reduce carcinogenic organisms (57).

## 7 CRISPR-Cas as a biomarker and therapeutic tool in GI cancer

CRISPR-Cas systems, beyond their bacterial immune function, are emerging as tools to detect and modulate microbial contributors to GI cancer (**Table 3**). CRISPR spacer and Cas gene profiles have been investigated primarily as genomic markers for bacterial strain discrimination and tracking, particularly in microbiome research (22, 58). For *H. pylori*, even incomplete CRISPR-like loci can facilitate high-resolution strain discrimination and characterization of genomic diversity (22, 58). Whether specific CRISPR profiles are associated with differences in virulence or gastric cancer risk remains uncertain and requires validation in well-characterized clinical cohorts. CRISPR-based genome editing also offers precision tools for microbiome engineering. Techniques such as CRISPR-Cas9 plasmids, phagemids, or nanoparticle delivery allow targeted killing or silencing of oncogenic bacteria, while sparing beneficial microbes (54, 59, 60). Combining CRISPR with engineered bacteriophages enhances this precision by using phages as vectors to carry programmable CRISPR payloads that eliminate harmful strains or resistance genes (59–62).

**Table 3:** CRISPR-Cas applications in gastrointestinal cancer risk.

Application	Mechanism	Potential benefit
Biomarker (CRISPR Profiles) (22)	Analysis of CRISPR spacers or Cas genes to distinguish bacterial strains	Identifies dysbiosis or virulent phenotypes
CRISPR Diagnostics (64–66)	Cas12/13 assays allow sensitive, specific detection of cancer biomarkers (nucleic acids, proteins, vesicles)	Enables rapid, sensitive, non-invasive screening
Microbiome Editing (54, 59, 60)	CRISPR delivery systems target pathogenic genomes	Removes oncogenic or resistant microbes while sparing commensals
Immunotherapy Enhancement (55, 84)	Engineers T cells (e.g., CAR-T) through targeted gene edits that boost antitumor activity	Improves the efficacy of adoptive therapies and minimizes adverse events
Gene Therapy (55, 84, 85)	Guide RNAs and Cas nucleases are used to precisely repair mutations or disrupt oncogenes through targeted genome editing	Repairs mutations, knocks out oncogenes, enables personalized therapies
CRISPR-Enhanced Phage Therapy (59–62)	Phages engineered with CRISPR for targeted lysis	Combines phage specificity with gene-level precision

CRISPR, clustered regularly interspaced short palindromic repeats; Cas, CRISPR-associated protein; Cas12/13, CRISPR-associated protein 12/13.

On the diagnostic front, CRISPR systems (e.g., Cas12 and Cas13) enable rapid, sensitive detection of microbial or host biomarkers associated with GI cancer (63). Promising targets include methylated cancer markers (like SEPT9) or oncogenic gene variants using point-of-care platforms that may eventually be deployable in stool or breath assays (64–66). While these CRISPR-based approaches are transformative, challenges remain- data standardization, delivery mechanisms to the gut or stomach, and clinical validation. Still, the triad of CRISPR as a biomarker, precision antimicrobial, and rapid diagnostic tool offers a powerful framework to tackle GI cancer risk from multiple angles.

## 8 Knowledge gaps and challenges in CRISPR-Cas research in gut microbiota and Gl cancers

While CRISPR-Cas systems have revolutionized microbial genetics and therapeutic development, their roles within the human gut microbiota and particularly in the context of GI cancer remain poorly understood. One critical knowledge gap lies in the limited characterization of CRISPR loci in *H. pylori*, a major gastric carcinogen. Although *H. pylori* is known for its genomic plasticity and adaptability, studies identifying and classifying CRISPR-Cas systems in this pathogen are sparse, inconsistent, and often limited to in silico predictions without functional validation (67, 68). This hinders the understanding of immune evasion and the potential of genome editing in gastric cancer. Therefore, functional annotation and expression profiling of *H. pylori* strains are essential to clarify CRISPR-Cas activity, its contribution to virulence and resistance, and its relevance as a therapeutic target.

Another significant complication is the technical difficulty of detecting functional CRISPR activity within complex microbial communities such as the gut. Metagenomic sequencing often captures CRISPR arrays or Cas gene homologs but does not confirm whether these systems are transcriptionally active or capable of adaptive immunity *in vivo*. For this, the use of metatranscriptomics and Cas protein–based detection platforms will be crucial to establish functional activity and improve resolution in community-wide analyses.

In addition, off-target effects represent a significant barrier in the clinical application of CRISPR-based microbiota editing or gene therapy. The gut environment contains high microbial diversity and genetic redundancy, making it difficult to design single-guide RNAs (sgRNAs) that are both specific and effective. Inadvertent cleavage of commensal or beneficial microbes may worsen dysbiosis or trigger unintended immune responses (69). This highlights the need for better off-target prediction tools. Moreover, many studies still rely on *in vitro* models or gnotobiotic mice, which lack the microbial and immunological complexity of the human GI tract.

Perhaps most importantly, there is a paucity of longitudinal human studies directly evaluating whether endogenous CRISPR-Cas activity in gut microbes is associated with GI cancer risk or progression. Most current evidence is cross-sectional, based on genomic or bioinformatic profiles, or derived from experimental systems, and therefore cannot establish temporal or causal relationships. In particular, CRISPR-like profiles in *H. pylori* have primarily been evaluated for strain discrimination rather than as validated predictors of gastric cancer risk (22). Long-term, multi-omics cohort studies integrating CRISPR activity with cancer biomarkers, immune profiles, microbiota composition, and clinical outcomes are therefore needed to determine whether observed associations have biological and clinical significance (70).

## 9 Strengths and limitations of the review

This review synthesizes evidence across mechanistic studies, microbial ecology, and translational applications, and offers a multi-level perspective on the microbiota–cancer axis. It uniquely emphasizes the underexplored relationship between CRISPR-associated genomic features, *H. pylori* genomic plasticity, and GI cancer research, while recognizing that direct evidence linking endogenous CRISPR activity to cancer risk remains limited. Furthermore, the dual role of CRISPR has been highlighted as an intrinsic microbial regulator and an engineered tool for diagnostics and therapeutics, framing these systems within gut dysbiosis, phage–bacteria dynamics, and cancer biomarkers to provide a holistic roadmap for future research (71). However, a major limitation of the review is the paucity of longitudinal human data which may hamper the efforts to distinguish correlation from causation in CRISPR–cancer interactions.

## 10 Future directions

The integration of CRISPR-Cas technologies into microbiome-cancer research signals a transformative opportunity for precision medicine, particularly in GI cancers. As multi-omics pipelines—encompassing genomics, transcriptomics, and metabolomics—become central to understanding cancer-microbiome interactions, CRISPR-Cas systems offer precise tools for dissecting microbial contributions to oncogenesis (72). When combined with omics technologies, it allows for comprehensive analysis of microbial communities and their interactions with the tumor microenvironment (72). For instance, researchers have developed efficient CRISPR-Cas-based genome editing tools for human gut Bacteroides species that enables markerless gene deletion and insertion (57). Future studies should focus on standardizing these approaches to ensure reproducibility across diverse microbial ecosystems, integrating data from metagenomics and metabolomics to map microbial-host interactions comprehensively.

Recent research shows the potential of targeting specific bacteria species for metabolite production as a promising approach in CRC prevention and treatment. CRISPR-based microbiome engineering may provide a more precise approach to modulating microbial metabolite production. In particular, targeted editing of metabolic pathways in gut bacteria could potentially alter the production of SCFAs such as butyrate, propionate, and acetate, which have important roles in epithelial homeostasis, immune regulation, and CRC biology. These metabolites can induce epigenetic modifications that may inhibit CRC progression (73, 74). CRISPR-based editing of Bacteroides species has enabled functional manipulation of genes involved in carbohydrate utilization and microbial community interactions, with demonstrated effects on community-level butyrate production (74). Conjugated linoleic acid metabolites produced by gut bacteria, such as *Bifidobacterium* and *Lactobacillus* species, have demonstrated anticarcinogenic effects in CRC by influencing inflammation, proliferation, and apoptosis pathways (75). More recent work using CRISPR-Cas has also identified functional carbohydrate-utilization pathways in *Bacteroides dorei*, illustrating how these tools can be used to investigate nutrient utilization and microbial ecology in the gut (76). Furthermore, strategies to modulate the gut microbiome, such as microbiome-based precision nutrition approaches and fecal microbial transplantation are being explored as potential interventions for CRC prevention and treatment (75, 77), stressing the necessity of further research into microbial metabolites and their effects on CRC.

The exploration of synthetic CRISPR-Cas systems as microbiota modulators represents an innovative frontier in microbiome research and therapeutics. These systems enable precise genome editing and control of microbial communities, with promising applications in personalized medicine and the treatment of dysbiosis-related conditions such as metabolic disorders (78, 79). Integrating phage recombination proteins and using non-canonical nucleotides such as 2-aminoadenine in crRNA further improve editing outcomes with minimal off-target effects (80).

In addition, clinical trials that investigate the use of CRISPR-based approaches to modulate microbiota or the host are required in order to apply this technology to the clinic. Translational research using tissue, blood, and imaging biomarkers has enabled better insight into treatment sensitivity and resistance mechanisms of GI cancers by incorporating tissue, plasma, and radiological biomarkers to inform next-generation trial design (81). Safety, tolerance, stability of the microbiota, and any off-target effects should be assessed during early-stage trials, whereas efficacy trials should evaluate the effectiveness of the treatment, including *H. pylori* eradication, treatment efficacy, relapse, and alteration of microbial flora and metabolites. For microbiome-based treatments, safety concerns regarding adverse effects on commensal microorganisms, development of antimicrobial resistance, GI inflammation, and ecological consequences for the microbiome should be monitored.

## 11 Conclusion and recommendations

CRISPR-Cas systems are important regulators of microbial ecology and promising engineered tools for cancer diagnostics and therapeutics. In GI cancer research, their potential relevance arises from their effects on microbial evolution, gene transfer, strain diversity, and microbiota interactions; however, direct evidence that endogenous CRISPR-Cas activity itself alters GI cancer risk remains limited. In *H. pylori*, current evidence primarily supports the use of CRISPR-like genomic features for strain discrimination and epidemiological characterization rather than as established determinants or predictors of gastric carcinogenesis.

Future investigations should focus on longitudinal human cohort studies to establish causal links between microbial CRISPR activity and cancer outcomes, along with cross-disciplinary collaboration between microbiologists, bioengineers, oncologists, and computational scientists. The development of CRISPR-based biomarkers for early detection of high-risk microbial signatures and cancer-specific alterations should be pursued in parallel. To ensure safety and specificity, optimizing delivery systems (phage vectors, nanoparticles, engineered probiotics) and integrating CRISPR with multi-omics platforms within clinical trials will be key to advancing precision microbiome editing into oncology applications. Ultimately, continued advances in CRISPR-Cas biology and engineering have the potential to transform the prevention, early detection, and treatment of GI cancers by enabling more precise microbiome-based diagnostics and targeted therapeutic strategies. Harnessing CRISPR-Cas systems could therefore establish a new paradigm in precision GI oncology, bridging fundamental microbial biology with clinically translatable interventions.
